# 3D-Printed Microinjection Needle Arrays via a Hybrid DLP-Direct Laser Writing Strategy

**DOI:** 10.1002/admt.202201641

**Published:** 2023-02-05

**Authors:** Sunandita Sarker, Adira Colton, Ziteng Wen, Xin Xu, Metecan Erdi, Anthony Jones, Peter Kofinas, Eleonora Tubaldi, Piotr Walczak, Miroslaw Janowski, Yajie Liang, Ryan D. Sochol

**Affiliations:** Department of Mechanical Engineering, University of Maryland, College Park, MD 20742, USA; Maryland Robotics Center, University of Maryland, College Park, MD 20742, USA; Institute for Systems Research, University of Maryland, College Park, MD 20742, USA; Department of Mechanical Engineering, University of Maryland, College Park, MD 20742, USA; Maryland Robotics Center, University of Maryland, College Park, MD 20742, USA; Institute for Systems Research, University of Maryland, College Park, MD 20742, USA; Department of Mechanical Engineering, University of Maryland, College Park, MD 20742, USA; Department of Mechanical Engineering, University of Maryland, College Park, MD 20742, USA; Department of Chemical and Biomolecular Engineering, University of Maryland, College Park, MD 20742, USA; Department of Mechanical Engineering, University of Maryland, College Park, MD 20742, USA; Maryland Robotics Center, University of Maryland, College Park, MD 20742, USA; Institute for Systems Research, University of Maryland, College Park, MD 20742, USA; Department of Chemical and Biomolecular Engineering, University of Maryland, College Park, MD 20742, USA; Department of Mechanical Engineering, University of Maryland, College Park, MD 20742, USA; Maryland Robotics Center, University of Maryland, College Park, MD 20742, USA; Institute for Systems Research, University of Maryland, College Park, MD 20742, USA; Program in Image Guided Neurointerventions, Department of Diagnostic Radiology and Nuclear Medicine, University of Maryland School of Medicine, Baltimore, MD 21201, USA; Program in Image Guided Neurointerventions, Department of Diagnostic Radiology and Nuclear Medicine, University of Maryland School of Medicine, Baltimore, MD 21201, USA; Program in Image Guided Neurointerventions, Department of Diagnostic Radiology and Nuclear Medicine, University of Maryland School of Medicine, Baltimore, MD 21201, USA; Department of Mechanical Engineering, University of Maryland, College Park, MD 20742, USA; Maryland Robotics Center, University of Maryland, College Park, MD 20742, USA; Institute for Systems Research, University of Maryland, College Park, MD 20742, USA; Fischell Department of Bioengineering, University of Maryland, College Park, MD 20742, USA; Robert E. Fischell Institute for Biomedical Devices, University of Maryland, College Park, MD 20742, USA

**Keywords:** 3D printing, additive manufacturing, digital light processing, direct laser writing, microneedles

## Abstract

Microinjection protocols are ubiquitous throughout biomedical fields, with hollow microneedle arrays (MNAs) offering distinctive benefits in both research and clinical settings. Unfortunately, manufacturing-associated barriers remain a critical impediment to emerging applications that demand high-density arrays of hollow, high-aspect-ratio microneedles. To address such challenges, here, a hybrid additive manufacturing approach that combines digital light processing (DLP) 3D printing with “ex situ direct laser writing (*es*DLW)” is presented to enable new classes of MNAs for fluidic microinjections. Experimental results for *es*DLW-based 3D printing of arrays of high-aspect-ratio microneedles—with 30 μm inner diameters, 50 μm outer diameters, and 550 μm heights, and arrayed with 100 μm needle-to-needle spacing—directly onto DLP-printed capillaries reveal uncompromised fluidic integrity at the MNA-capillary interface during microfluidic cyclic burst-pressure testing for input pressures in excess of 250 kPa (*n* = 100 cycles). Ex vivo experiments perform using excised mouse brains reveal that the MNAs not only physically withstand penetration into and retraction from brain tissue but also yield effective and distributed microinjection of surrogate fluids and nanoparticle suspensions directly into the brains. In combination, the results suggest that the presented strategy for fabricating high-aspect-ratio, high-density, hollow MNAs could hold unique promise for biomedical microinjection applications.

## 1. Introduction

Microinjection technologies underlie a diversity of biomedical applications, such as in vitro fertilization, intraocular injection, therapeutic drug and vaccine delivery, developmental biology, and transgenics.^[1–4]^ Historically, microinjection protocols have relied on using a single hollow microneedle to deliver target substances (e.g., cells, DNA, RNA, micro/nanoparticles) to a singular location of interest.^[5–7]^ Recently, however, alternatives in the form of microneedle arrays (MNAs) have garnered increasing interest due to a wide range of benefits over their single-needle counterparts, including the ability to rapidly deliver target material over a large, distributed area, which has proven to be particularly beneficial for transdermal and intradermal drug delivery. ^[8–11]^ Despite the significant potential of MNAs for microinjection applications, the majority of current MNA developments are founded on solid (e.g., coated and/or dissolvable) microneedles that are inherently incompatible with active fluidic microinjection protocols.^[12–14]^ This focus on solid MNAs is, in part, due to the considerable challenges associated with manufacturing arrays comprising hollow microneedles at small scales. Specifically, although researchers have demonstrated that conventional clean room-based micromachining approaches can be adapted to fabricate arrays of hollow microneedles,^[15–17]^ such protocols can be exceedingly time-, cost-, and labor-intensive, while restricting the architectures of the microneedles to low-aspect-ratio “2.5D” geometries.^[18–20]^ The geometric limitations, in particular, represent a significant barrier to extending the benefits of MNAs to emerging microinjection applications, such as for treatments of neurological conditions.

One example of such a treatment in which MNAs could potentially offer benefits over single-needle injection strategies is stem cell therapy (SCT). A crucial obstacle to the clinical efficacy of SCT is the poor viability of stem cells following delivery into the brain.^[21–23]^ One challenge associated with conventional needles is cell crowding at the injection site due to the high concentrations of donor cells (e.g., up to 100 000 cells μL^−1^),^[24,25]^ which can lead to large cell spheroids with undesirable conditions (e.g., decreased access to O_2_ and nutrients for interior cells) that contribute to the low survival rates of implanted stem cells.^[26–29]^ It is possible that simultaneous, distributed cell delivery via MNAs could provide novel means to improve cell survival rates by reducing cell crowding; however, no MNA yet exists to enable such studies. For instance, even in the case of mice—a widely used disease model^[30]^ with a relatively shallow (≈1 mm) cerebral cortex compared to other animal models^[31]^—the ability to penetrate into the cerebral cortex for therapeutics delivery would necessitate hollow microneedles that not only comprise outer diameters (ODs) on the order of tens of micrometers but also include heights in excess of 500 μm. Consequently, new strategies for manufacturing MNAs composed of such high-aspect-ratio, hollow microneedles are in critical demand.

Additive manufacturing (or colloquially, “3D printing”) technologies offer distinctive benefits for applications that require a high degree of geometric control in component fabrication^[32−34]^ Previously, researchers have demonstrated a wide range of 3D printing techniques for the fabrication of needle arrays at various scales. For example, at larger scales, Derakhshandeh et al. used extrusion-based 3D printing (e.g., “direct ink writing”) to manufacture arrays of hollow, millimeter-scale needles for drug delivery,^[35]^ which facilitated enhanced wound healing.^[36]^ For mesoscale needles, however, the print speed and geometric limitations of extrusion-based methods at smaller scales^[37–39]^ have motivated investigators to instead focus on fabricating MNAs via vat photopolymerization approaches, such as stereolithography and digital light processing (DLP) 3D printing^[40–42]^ Unfortunately, these printing techniques are poorly suited for printing hollow MNAs that comprise needles with sub-100 μm ODs, which has led to increasing interest in the use of “direct laser writing (DLW)” for such cases.

DLW entails scanning a femtosecond pulsed IR laser in a point-by-point, layer-by-layer manner to selectively crosslink a photocurable material in target locations via two-photon (or multiphoton) polymerization to ultimately produce 3D objects comprising cured photomaterial with feature resolutions down to the 100 nm range.^[43–46]^ Previously, researchers have demonstrated the utility of using DLW to print MNA master molds, which can then be used to replicate solid MNAs with drug coatings^[47–50]^ or solid MNAs that are fully dissolvable^[51,52]^ Additionally, Rad et al. reported the use of DLW to print molds and MNAs directly that include open (i.e., unenclosed) side channels^[53–55]^ For realizing hollow microneedles that are a requisite for microinjection applications, one key challenge inherent to the submicrometer-scale resolution of the DLW-printing volume element (i.e., “voxel”) is that it is ill suited for constructing the larger macro-to-microinterfaces (e.g., input ports) required for delivering fluids to the needles.^[56,57,92]^ To avoid the undesirable costs and time associated with fabricating macro-to-microinterfaces in their entirety via DLW,^[58]^ researchers have instead DLW-printed hollow singular microneedles (aspect ratios ≈4–5)^[59]^ and MNAs (aspect ratios ≈2–5)^[60]^ as isolated entities, and then used adhesives (e.g., glue) to manually connect the printed components to macroscale fluidic interfaces. Trautmann et al. bypassed such protocols by employing a fabrication methodology that combines femtosecond laser irradiation, annealing, grinding, and polishing to produce microchips with external openings, and then DLW-printing truncated coneshaped MNAs (aspect ratios ≈1.3–3) directly onto the chips.^[61]^ In contrast to the aforementioned approaches, printing MNAs directly onto fluidic connectors (e.g., at the end of capillaries) would overcome many of the current interface-associated barriers to MNA utility. Furthermore, to our knowledge, no report yet exists (for conventional or additive manufacturing-based approaches) in which MNAs are fabricated with hollow, high-aspect-ratio (e.g., ≥10) microneedles with microscale ODs (e.g., <100 μm) and high array densities (e.g., ≤100 μm needle-to-needle spacing) relevant to emerging microinjection applications, such as the delivery of therapeutic fluidic payloads directly into brain tissue.

**Figure 1.**
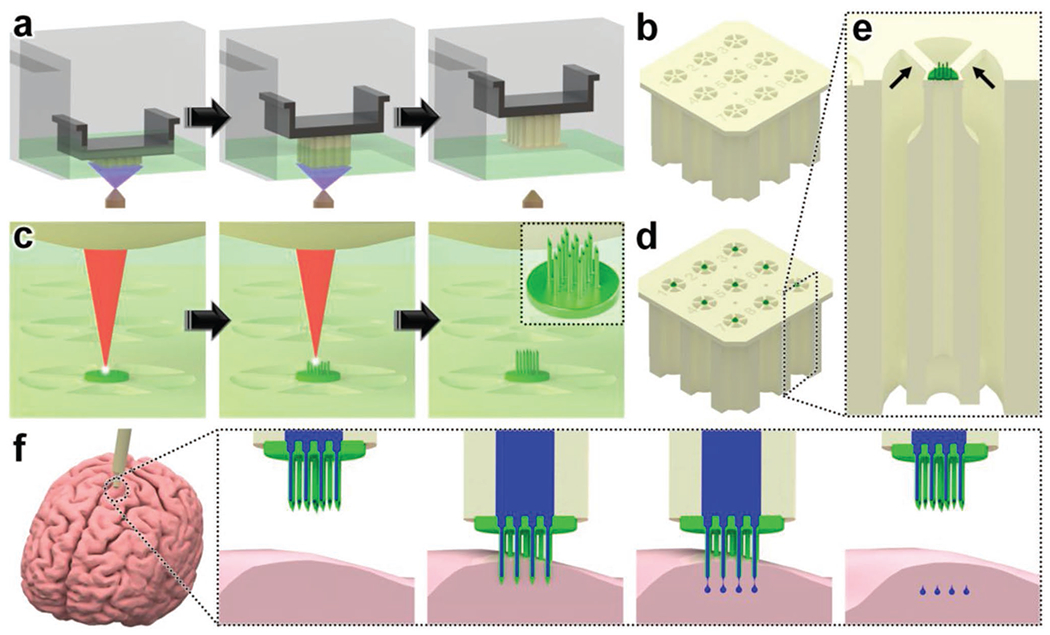
Conceptual illustrations of the hybrid additive manufacturing strategy for 3D microprinting hollow, high-aspect-ratio microneedle arrays (MNAs) for microinjection applications. a,b) Digital light processing (DLP)-based 3D printing of batch capillaries. a) A liquid-phase photocurable material is UV-crosslinked in designated locations in a layer-by-layer manner to produce a batch of arrayed capillaries comprising cured photomaterial. b) The DLP-printed batch of prealigned capillaries following the development process. c–e) “Ex situ direct laser writing (*es*DLW)” of MNAs directly atop—and fluidically sealed to—each DLP-printed capillary. c) A femtosecond pulsed IR laser is scanned to selectively initiate two-photon polymerization of a liquid-phase photocurable material in a point-by-point, layer-by-layer manner to produce MNAs comprising cured photomaterial. d) A batch array of MNA-capillary assemblies following the DLW-associated development process. e) Individual MNA-capillary assemblies within the array can be released on demand by manually severing the supporting structures (arrows). f) Example application of integrating MNA-capillary assemblies with nanoinjector systems to facilitate MNA-mediated simultaneous, distributed microinjections of target fluidic substances/suspensions into brain tissue.

In this work, we introduce a novel hybrid additive manufacturing strategy that entails using DLP 3D printing to fabricate batches of capillaries in set positions (Figure 1a,b), and then employing an “ex situ DLW (*es*DLW)” approach to DLW-print hollow, high-aspect-ratio, high-density MNAs directly onto— and notably, fluidically sealed to—the DLP-printed capillaries (Figure 1c,d). Thereafter, individual MNA-capillary assemblies can be selectively released by disrupting the connections to the batch (Figure 1e, arrows) and then interfaced with injector systems for microinjection applications. As an exemplar, we investigate the utility of the MNAs for performing microinjections into brain tissue (Figure 1f) by using excised mouse brains to not only evaluate MNA penetration into and retraction from the tissue with respect to microneedle integrity but also explore the efficacy of MNA-mediated delivery of microfluidic cargo (e.g., aqueous fluids and nanoparticle suspensions) into brain tissue ex vivo.

## 2. Results and Discussion

### 2.1. Hybrid Additive Manufacturing of Hollow MNAs

The presented hybrid additive manufacturing strategy consists of two fundamental stages: i) DLP 3D printing of batch arrays of capillaries and ii) *es*DLW-based printing of the MNAs directly atop each capillary. DLP 3D printing is a vat photopolymerization approach in which a DLP projector is used to UV-crosslink a liquid-phase photocurable material in designated locations in a layer-by-layer manner to ultimately produce 3D objects composed of cured photomaterial.^[62]^ Here, we leveraged DLP 3D printing to fabricate batches of arrayed capillaries in a single print run to overcome several drawbacks of recent *es*DLW approaches for printing 3D micro/nanostructured objects onto mesoscale fluidic components, such as micropiston-based microgrippers^[63]^ and liquid biopsy systems^[64]^ onto fluidic capillaries. First, the geometric control afforded by DLP 3D printing allows for each capillary to be designed with a variable OD to match the dimensions of the capillary base to those of the desired injector system. This capillary-specific geometric customization capability obviates the need for additional fluidic adapters and/or sealants (e.g., glues) often required to couple the mesoscale capillaries to macroscale fluidic equipment (e.g., injector systems).^[63–65]^ Second, the outer dimensions of the batch array can be designed to support facile loading into the DLW 3D printer, which eliminates the time, labor, and costs associated with manufacturing and employing custom-built capillary holders typically needed for *es*DLW approaches.^[63–65]^ Lastly, the ability to print all of the capillaries in predefined array locations—with uniform surface positions and rotational orientations—addresses critical deficits associated with the use of custom-built capillary holders that rely on undesired manual (e.g., by hand and/or eye) alignment protocols for each individual capillary.^[65]^

**Figure 2.**
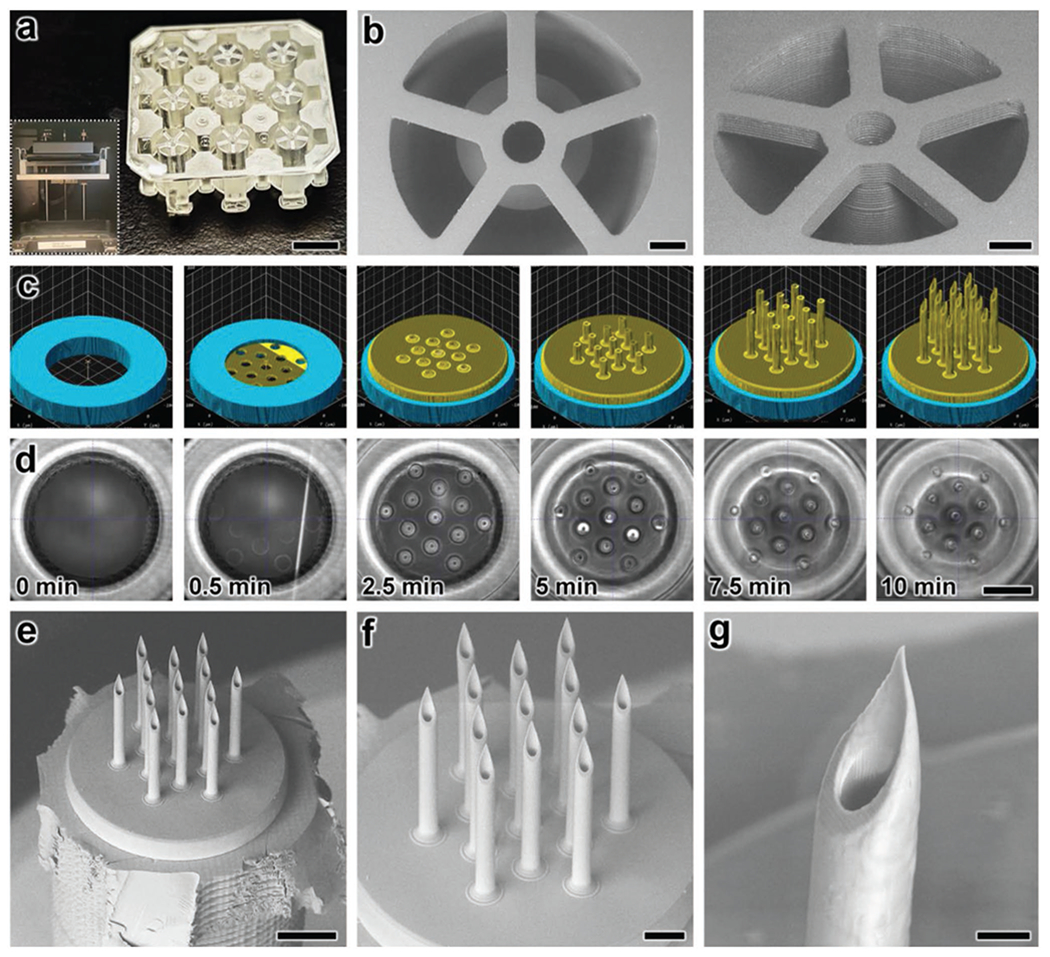
Fabrication results for DLP 3D printing of batch arrays of capillaries and *es*DLW-based printing of MNAs. a,b) DLP prints of batch arrays of capillaries. a) Photograph of a complete batch with nine arrayed capillaries. Scale bar = 5 mm. Inset shows two batches attached to the build plate directly after DLP 3D printing (see Movie S1 in the Supporting Information). b) Low-vacuum scanning electron microscopy (SEM) images of a representative DLP-printed capillary attached to the batch via five connecting structures. Scale bars = 500 μm. c,d) The *es*DLW approach for printing MNAs directly onto DLP-printed capillaries in a single print run. c) Computer-aided manufacturing (CAM) simulations and d) corresponding images of the *es*DLW printing process. Scale bar = 250 μm (see Movie 2 in the Supporting Information). e–g) Low-vacuum SEM images of representative fabrication results showing: e) an *es*DLW-printed MNA atop a DLP-printed capillary following release from the batch array (see Movie S3 in the Supporting Information); f) a magnified view of the MNA; and g) a magnified view of a single microneedle tip in the array. Scale bars = e) 250 μm, f) 100 μm, and g) 25 μm.

For DLP 3D printing of the batch capillary arrays, we used a Miicraft M50 microfluidics DLP 3D printer (CADworks3D, Toronto, ON, Canada) to fabricate two batches (i.e., 18 capillaries in total) per print run, which corresponded to a total print time of less than 45 min (Movie S1, Supporting Information). To enable direct integration with the nanoinjector system (MO-10, Narishige International USA, Inc., Amityville, NY), we designed each capillary with a consistent inner diameter (ID) of 650 μm, but with a variable OD that was set at 1.2 mm for the top 1.5 mm and then gradually increased to 2.4 mm for the remainder of the 10 mm length of the capillary (Figure S1, Supporting Information). Fabrication results revealed effective construction of the arrayed capillaries—each attached to the batch via five connecting structures (400 μm in width and depth; 1.5 mm in length) (Figure 2a,b). In addition, the outer dimensions of the overall batch resolved such that the print could be readily loaded into the multi-DiLL holder of the DLW system (Photonic Professional GT2, Nanoscribe GmbH, Germany) (Figure S2, Supporting Information) to facilitate *es*DLW-based 3D printing.

We designed the MNAs to include identical hollow microneedles—each with an ID of 30 μm, an OD of 50 μm, and a height of 550 μm—with needle-to-needle spacing of 100 μm (Figure S3, Supporting Information). For the *es*DLW printing process, we initiated the print with 50 μm of overlap with the top surface of the capillary to ensure bonding at the interface. Computer-aided manufacturing (CAM) simulations and brightfield images of a corresponding *es*DLW process for printing the MNA directly onto a DLP-printed capillary are presented in Figure 2c,d, respectively (*see also* Movie S2, Supporting Information). The total *es*DLW printing process was completed in ≈10 min. Following development, we retrieved target MNA-capillary assemblies from the batch by manually severing the five connecting structures (Movie S3, Supporting Information). Images of the released MNA-capillary assemblies captured using low-vacuum scanning electron microscopy (SEM) revealed effective alignment and integration of the *es*DLW-printed MNAs with the DLP-printed capillaries, without any visible signs of physical defects along the MNA-capillary interface (Figure 2e). In addition, images of the *es*DLW-printed MNA and needle tips suggest that the manual release process did not appear to affect MNA integrity (Figure 2f,g).

### 2.2. In Silico and In Vitro Investigations of MNA Mechanical Performance

**Figure 3.**
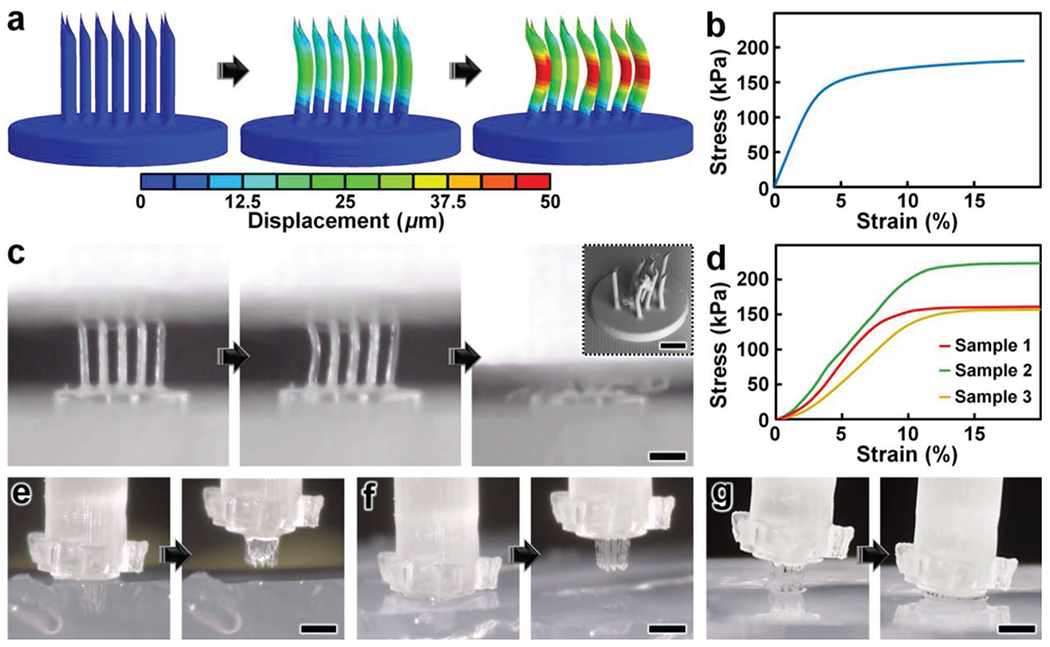
Numerical and experimental results for MNA mechanical characterizations. a,b) Finite element analysis (FEA) results for a) microneedle deformations and b) stress-strain curve corresponding to MNA mechanics under compressive loading conditions. c,d) Experimental results for MNA compression testing. c) Sequential images of the MNA during axial compression test. Inset shows an SEM image of an MNA following compressive failure. Scale bars = 250 μm (see Movie S4 in the Supporting Information). d) Stress–strain curve generated from compressive loading experiments (*n* = 3 MNAs). e–g) Sequential images of representative MNA penetration and retraction operations corresponding to hydrogels with agarose concentrations of: e) 2.4%, f) 5%, and g) 10%. Scale bars = 500 μm (see Movie S5 in the Supporting Information).

The critical first steps of MNA-based microinjection protocols involve the effective puncture and penetration into a target medium (e.g., biological tissue), which can impart significant mechanical forces on the microneedles.^[66]^ Thus, the potential utility of MNAs is predicated on their ability to successfully withstand such mechanical loading conditions. To evaluate this capability for the *es*DLW-printed high-aspect-ratio MNAs, we employed both numerical and experimental approaches to elucidate the mechanical performance of the MNAs. We performed finite element analyses (FEA) to provide insight into the mechanical failure behavior of the MNAs when subjected to a compres sive load applied longitudinally with respect to the needles. The simulation results revealed that each arrayed microneedle exhibited a buckling-like deformation with the largest displacements observed around the midpoint of the heights; however, needles positioned in the outer region (i.e., the needles radially arrayed farthest from the center of the MNA) displayed larger deformations compared to those located in the more central array positions (Figure 3a). This behavior arises from the load distribution caused by the disc-like base of the MNA, which deforms more in its central region than its peripherical region, thereby allowing the centrally located microneedles to rigidly displace more in the axial direction than their outer-region counterparts. According to the stress-strain curve generated from the FEA compressive loading simulations (Figure 3b), the overall MNA exhibited an effective Young’s Modulus (*E*) of 4.31 MPa and yield strength (*σγ*) of 135 kPa. We also numerically modeled MNA mechanics associated with puncture into the brain tissue. By characterizing the nonlinear response at the interface between the tips of the microneedles and the brain substrate, we found that the forces associated with the needles located in the outer region were larger than those in the central regions (Figure S4, Supporting Information), which is in agreement with the compressive loading analyses (Figure 3a).

To experimentally examine the mechanical performance of the *es*DLW-printed MNA, we conducted two sets of puncture and penetration-associated studies. First, we performed axial compression tests with *es*DLW-printed MNAs (*n* = 3), which revealed buckling-type deformations of the microneedles with increasing loading until complete mechanical failure (Figure 3c and Movie S4, Supporting Information). From SEM images of MNAs following compressive testing, we observed several cases of complete fracture, but the majority of the arrayed microneedles remained intact with the caveat that the tips and the overall shapes of the needles exhibited plastic deformation (Figure 3c, inset). Quantified results for the stress-strain relationships for the *es*DLW-printed MNAs revealed an average *E* of 2.12 ± 0.35 MPa and *σγ* of 155 ± 30 kPa (Figure 3d). Although these results provide insight into the upper boundaries of mechanical loading, compression testing using an impenetrable plate is limited in its direct relevance to microinjection applications that rely on microneedle penetration into a target medium. Thus, we also investigated the capacity for the *es*DLW-printed MNAs to puncture and penetrate into surrogate hydrogels with increasing concentrations of agarose that correspond to varying degrees of biologically relevant stiffness. In particular, we performed experiments with agarose concentrations of: i) 1.2% (*E =* 12.8 ± 1.1 kPa), which would support penetration into liver and breast tissue; ii) 2.4% (*E =* 275 ± 1.0 kPa), which is relevant to brain, heart, kidney, arterial, and prostate tissue; and iii) both 5% (*E =* 223 ± 14 kPa) and 10% (*E =* 268 ± 31 kPa), which are relevant to cartilage tissues (Figure S5, Supporting Information).^[67–70]^ Experimental results revealed that the MNA successfully penetrated into the 1.2%, 2.4%, and 5% agarose gels; however, we observed buckling of the microneedles and failure to penetrate into the 10% agarose gel (Figure 3e–g and Movie S5, Supporting Information). These results suggest that the *es*DLW-printed MNA is sufficient for penetration into brain tissue as well as a variety of other tissues (e.g., liver, breast, heart, kidney, arterial, and prostate tissues), but alternative photomaterials (with stronger mechanical properties) and/or microneedles with geometrically enhanced strength (e.g., by increasing the OD) would be needed for microinjection applications involving target mediums with *E* in excess of 250 kPa.

### 2.3. In Vitro Microfluidic Interrogations of MNA-Capillary Interface Integrity

**Figure 4.**
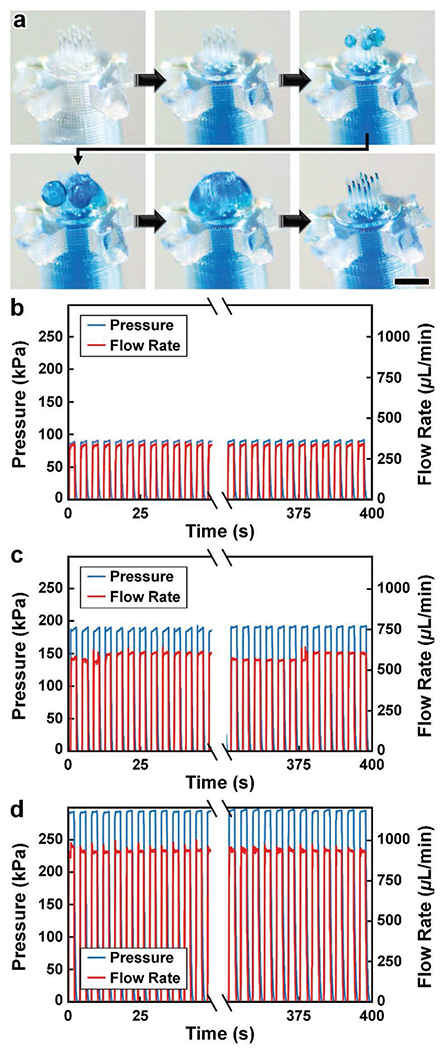
Experimental results for MNA microfluidic investigations. a) Sequential images during fluidic infusion. Scale bar = 500 μm (see Movie S6 in the Supporting Information). b–d) Quantified results for representative cyclic burst-pressure experiments (*n* = 100 cycles) corresponding to input pressures targeting: b) 100 kPa, c) 200 kPa, and d) 300 kPa.

One of the most catastrophic failure modes for *es*DLW-based prints—whether for optical,^[71]^ photonic,^[72]^ Mechanical,^[73]^ or fluidic ^[63–65]^ structures—is the potential for the DLW-printed objects to detach from the meso/macroscale components on which they are additively manufactured. For biomedical MNA applications, the consequences of this type of failure could be particularly serious, such as an MNA detaching from the capillary while embedded in brain tissue following microinjection. To investigate the potential for this failure mode and, in turn, provide insight into the mechanofluidic integrity of the interface between the *es*DLW-printed MNAs and the DLP-printed capillaries, we performed microfluidic cyclic burst-pressure tests with the MNA-capillary assemblies. Initially, using an applied pressure set at 5 kPa, we gradually infused blue-dyed deionized (DI) water into the MNA-capillary assembly via the opposing end of the capillary (i.e., the side without the printed MNA) until the fluid began exiting the tips of the arrayed microneedles (Figure 4a and Movie S6, Supporting Information). Thereafter, we performed separate sets of cyclic burst-pressure experiments (*n* = 100 cycles per experiment) corresponding to applied pressures set at 100, 200, and 300 kPa (Figure 4b–d). Throughout the burst-pressure testing, we monitored the MNA-capillary interface under brightfield microscopy for visible signs of undesired leakage phenomena (e.g., fluid exiting at any point along the interface rather than out of the tops of the microneedle tips); however, we did not observe any instances of such flow behavior. Similarly, quantified results of fluid flow through the MNA-capillary assembly recorded during the burst-pressure tests did not exhibit any indications of burst events—i.e., large increases in flow rates after a certain point, despite the applied pressure remaining constant—nor signs of gradual leakage phenomena associated with the flow rates increasing from pressure cycle to pressure cycle over the course of the experiment. Rather, the flow rate magnitudes corresponding to the applied input pressures remained consistent throughout the burst-pressure experiments (Figure 4b–d), suggesting uncompromised fluidic integrity of the MNA-capillary interface for all cases examined.

### 2.4. Ex Vivo Mouse Brain Studies of MNA Penetration, Microinjection, and Retraction Functionalities

**Figure 5.**
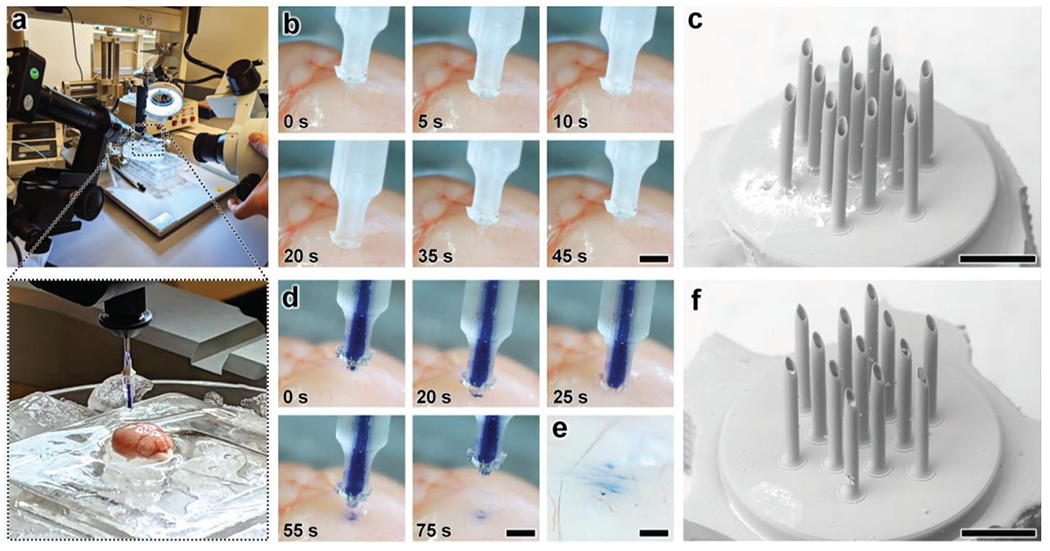
Experimental results for ex vivo MNA penetration, microinjection, and retraction operations using an excised mouse brain. a) Experimental setup including the MNA-capillary assembly interfaced with a custom-built nanoinjector and an excised mouse brain on ice. b,c) Brain tissue puncture and retraction results. b) Sequential images of MNA insertion into (≤20 s) and retraction from (≥20 s) the brain tissue. Scale bar = 1 mm (see Movie S7 in the Supporting Information). c) SEM image of the MNA after retraction from the brain tissue. Scale bar = 250 μm. d–f) MNA-mediated microinjection results. d) Sequential images of a representative MNA penetration, microinjection, and retraction process for a surrogate fluid (blue-dyed DI water) injected into brain tissue. Scale bar = 1 mm (see Movie S8, Supporting Information). e) Magnified view of the postinjection site. Scale bar = 250 μm. f) SEM image of the MNA following microinjection into the brain tissue. Scale bar = 250 μm.

As an exemplar with which to interrogate the penetration, microinjection, and retraction capabilities of the *es*DLW-printed MNAs, we excised brains with intact dura mater from euthanized 6-month-old male mice (Wild-type C57BL/6 J, Jackson Laboratory) for experimentation ex vivo (Figure 5a). We performed three sets of experiments to elucidate these fundamental MNA functionalities. First, we investigated the ability to execute penetration and retraction operations (but not fluidic microinjections) with the MNAs as critical measures of performance with respect to three potential failure modes that would critically limit the efficacy of the *es*DLW-printed MNAs: i) the sharpness of the tips of the microneedles—governed by the resolution of the DLW 3D printer—is insufficient to puncture the brain tissue without inducing significant deformation of the brain; ii) the mechanical properties of the high-aspect-ratio microneedles lead to buckling and/or fracture of the microneedles prior to effective penetration into the brain tissue; and/or iii) the forces during the penetration or retraction processes fracture the microneedles, causing microneedles (or fragments of microneedles) to remain embedded in the brain tissue after retraction completion. To facilitate the penetration and retraction studies, we interfaced each MNA-capillary assembly examined with a nanoinjector system fixed to a stereotactic frame as a means to enable precise position control while optically monitoring the MNA-brain tissue interactions. Experiments performed with three distinct MNA-capillary assemblies (*n* = 3 penetration and retraction operations for each distinct MNA-capillary assembly) revealed that the MNAs could successfully puncture the brain tissue within 1 mm of total displacement from initial contact and, importantly, without any visible signs of mechanical failure during any of the penetration or retraction operations (Figure 5b and Movie S7, Supporting Information). Images of the MNAs (captured after completion of the retraction process) corroborated these results, without any indications of microneedle-associated failure modes (e.g., buckling or fracture) or MNA detachment from the capillary (Figure 5c).

After validating the penetration and retraction capabilities, we then initially investigated the microinjection functionality of the MNAs based on the ability to deliver a surrogate microfluidic payload into the brain tissue. In this case, we preloaded the MNA-capillary assembly with blue-dyed (1.5% Evan’s Blue) DI water, and then interfaced the assembly with the nanoinjector (Figure 5a, expanded view) for control of both the MNA position and fluidic microinjection dynamics. Although the results for the cyclic microfluidic burst-pressure experiments performed in vitro (Figure 4b–d) suggested that the MNA-capillary interface should withstand the forces associated with microinjections into the brain tissue, we optically monitored the overall MNA-capillary assembly during the microinjection process for potential signs of undesired leakage via the interface. Akin to the tissue penetration and retraction studies, we used the stereotaxic frame to guide the descent of the MNA into the brain tissue (Figure 5d, top and Movie S8, Supporting Information). Following completion of the penetration process, we then used the pneumatically controlled nanoinjector to dispense the surrogate dyed fluid through the MNA-capillary assembly and, in turn, deliver the fluid into the brain tissue. Thereafter, we retracted the MNA from the brain (Figure 5d, bottom and Movie S8, Supporting Information), and then washed the surface of the injection site with phosphate buffered saline (PBS) to eliminate any residual surrogate fluid from the surface, such that the only remaining fluid was located beneath the tissue surface (Figure 5e). Throughout the microinjection process, we did not observe any undesired leakage phenomena (Movie S8, Supporting Information), with optical characterizations of the postinjection site indicating effective, distributed MNA-mediated delivery of the surrogate fluid well below the surface of the excised brain (Figure 5e). Furthermore, SEM images of the MNA-capillary assembly following tissue penetration, fluidic microinjection, and retraction revealed uncompromised structural integrity (Figure 5f).

**Figure 6.**
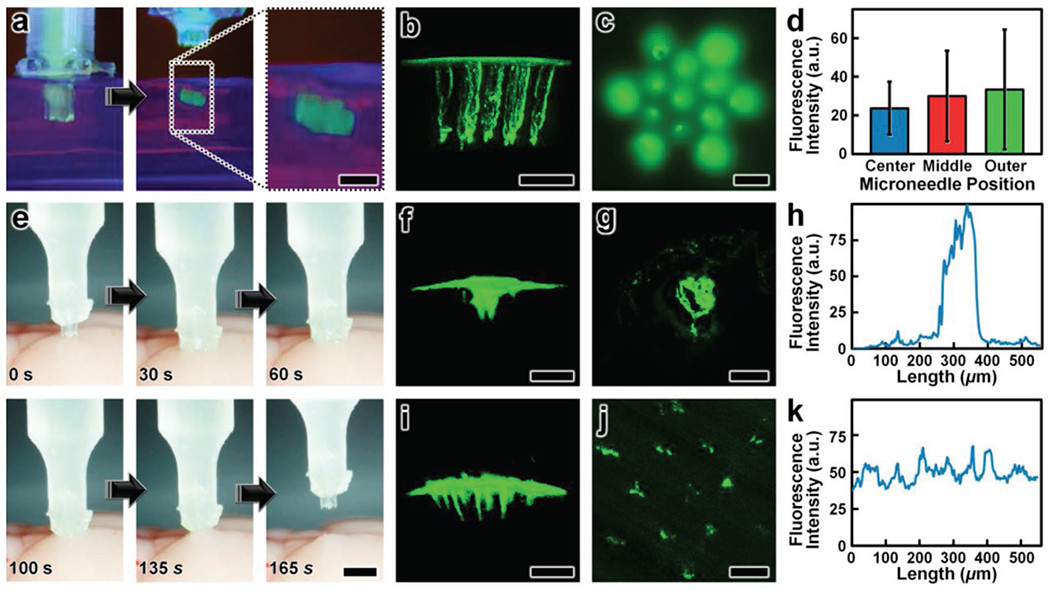
Experimental results for microinjections of fluorescent nanoparticles a–d) in vitro in 0.6% agarose gels and e–k) ex vivo using excised mouse brains. a) Sequential images of nanoparticle microinjection and retraction. Scale bar = 250 μm (see Movie S9 in the Supporting Information). b,c) Fluorescence images of the postinjection site captured using b) two-photon and c) widefield fluorescence microscopy. Scale bars = 250 μm. d) Mean fluorescence intensities of injection sites corresponding to microneedles in distinct array regions (*n* = 3 MNAs). Error bars = *S.D.* e) Sequential images of a representative MNA penetration, microinjection, and retraction process for a suspension of fluorescent nanoparticles injected into brain tissue. Scale bar = 1 mm (see Movie S10 in the Supporting Information). f–k) Postinjection results for fluorescent nanoparticles delivered via f–h) a conventional Hamilton 33G needle, and i–k) an *es*DLW-printed MNA. f,g,i,j) Fluorescence images of the postinjection site captured using two-photon fluorescence microscopy visualized in f,i) side and g,j) cross-sectional views. Scale bars = 250 μm. h,k) Quantified fluorescence intensities along the length of the corresponding cross-sectional views of the postinjection sites.

Lastly, we evaluated the microinjection performance of the *es*DLW-printed MNA compared to a conventional needle (Hamilton 33G) widely used for delivering therapeutics into brain tissue.^[74]^ In this case, we used a suspension of fluorescently labeled nanoparticles (100 nm in diameter) as the surrogate microfluidic payload. As an initial positive experimental control for the *es*DLW-printed MNA, we performed microinjections (*n* = 3 MNAs) of the nanoparticle suspension into 0.6% agarose gel in vitro (Figure 6a and Movie S9, Supporting Information) and visualized the particle distributions using two-photon (Figure 6b S10) and widefield fluorescence microscopy (Figure 6c). We observed injected nanoparticles corresponding to each microneedle in the array—which included one microneedle in the center of the array, six needles arrayed radially in a middle region (150 μm from the center), and six needles arrayed radially in an outer region (260 μm from the center)—but to determine if microneedle array position influenced injection behavior, we analyzed the fluorescence intensities associated with each arrayed needle. Quantified results revealed that the fluorescence intensities were statistically indistinguishable, with no discernable difference for the microneedle injection sites between the center and either the middle (*p* = 0.66) or outer regions (*p* = 0.61), nor between the middle and outer regions (*p* = 0.72) (Figure 6d). Thereafter, we performed microinjections of the nanoparticle suspension into excised mouse brains using both the conventional needle and the *es*DLW-printed MNA (Figure 6e and Movie S10, Supporting Information). Two-photon fluorescence images of the injection sites revealed stark differences in the nanoparticle distributions associated with each needle system. In the conventional needle case, the nanoparticles accumulated tightly within the single needle track (Figure 6f,g). For example, quantified fluorescence intensity results revealed that the majority of the fluorescence signal was detected within an ≈150 μm region (Figure 6h). In contrast, MNA-associated microinjection sites exhibited a more homogeneous distribution of injected nanoparticles over a larger area (Figure 6i,j)—with particles detected at sites corresponding to each arrayed microneedle—resulting in a more consistent fluorescence signal along the length of the injection site (Figure 6k). These results suggest that MNAs offer an effective means to distribute fluidic payloads more uniformly over a larger area compared to conventional single-needle systems. In combination, these experimental results for MNA penetration, surrogate fluid/suspension delivery, and retraction functionalities using an ex vivo mouse brain provide an important foundation for the utility of the presented hybrid DLP-DLW-enabled MNAs for microinjection applications.

## 3. Conclusions

Microneedle-based microinjection protocols are essential to wide-ranging fundamental research and clinical applications across biological and biomedical fields, with MNAs providing numerous benefits over their single-needle counterparts in many scenarios.^[75–77]^ Unfortunately, manufacturing-associated limitations have heretofore impeded researchers from leveraging the potential benefits of high-density MNAs comprising hollow, high-aspect-ratio microneedles at small length scales.^[78–80]^ In this work, we introduced the concept of using *es*DLW to 3D print MNAs directly atop DLP-printed capillaries in batch arrays and demonstrated this approach by fabricating arrays of 50 μm OD, 30 μm ID, 550 μm tall hollow microneedles with 100 μm needle-to-needle spacing. Because the presented strategy is founded on two additive manufacturing technologies, the inherent geometric versatility can be harnessed to tailor both the DLP-printed capillaries and the *es*DLW-based MNAs to target experimental setups and applications. For the DLP-printed capillary, the shape and size need not be uniform along the length of the capillary as is the predominant case for conventional and/or commercially available fluidic capillaries. Here, for instance, we designed the OD of the base of the capillary to yield facile, direct integration with the nanoinjector, thereby circumventing the need for additional fluidic adapters or sealants. Similarly, although the presented design for the *es*DLW-printed MNAs included identical microneedles with dimensions based on a specific exemplar—i.e., fluidic microinjection into the cerebral cortex of a mouse brain—the high architectural control and submicrometer-scale resolution of DLW can be leveraged to customize the size, shape, and position of each individual microneedle in an array as desired (Figure S4, Supporting Information). For example, future efforts could increase the microneedle heights substantially to target different regions of the brain and/or additional animal models. Conversely, while this work centered on printing hollow microneedles (with 30 μm IDs) to support fluidic delivery operations, given the recent developments for the utility of solid MNAs in other cases, the presented strategy could also be extended to print MNAs composed of solid microneedles, such as those fabricated using DLW-compatible biodegradable materials,^[81,82]^ or potentially hybrid MNAs that comprise both hollow and solid microneedles.

The presented strategy also provides an important foundation for future academic and industrial translation through four pathways. First, in contrast to prior *es*DLW efforts, DLP-printing of the batch arrays of fluidic capillaries allows for facile loading into the DLW 3D printer, obviating the need for custom-built capillary holders as well as the time- and labor-intensive protocols required to manually load each individual capillary into such holders. Furthermore, because each capillary is printed in a designated array position with specified orientations, the setup for initiation of the *es*DLW-printing process is minimized, which could provide a promising avenue to scalable and automated production. Second, although we employed a layer-by-layer DLP printer to manufacture the batch arrays of fluidic capillaries, numerous vat photopolymerization approaches could be used instead to increase production speed, including continuous liquid interface production to print parts in minutes^[83]^ and various volumetric 3D printing strategies to fabricate parts in tens of seconds.^[84–86]^ Third, for *es*DLW-based printing of the MNAs, while the voxel size remained constant throughout the printing process with a scan speed of ≈120 mm s^−1^, future efforts can harness recent advancements for state-of-the-art DLW printers that can not only dynamically tailor the size of the voxel to target features but also allow for scan speeds up to 1,250 mm s^−1^ (e.g., with 5× objective lens configurations) in order to dramatically enhance print efficiency and speed. Lastly, recent improvements in the available build area for commercial DLW printers could be extended to print multiple MNAs simultaneously in a single pass (in contrast to the serial MNA printing strategy reported here), which would further increase the attainable production volume.

The numerical and experimental mechanical characterizations of the *es*DLW-printed MNA suggest that, in addition to brain tissue, the MNA described in this work could be used to facilitate microinjections for a wide range of additional biological tissues, including those associated with the liver, breast heart, kidney, veins, arteries, and prostate.^[67–70]^ For future efforts based on different injection targets with higher stiffness (e.g., *E* > 250 kPa), however, the inability of the presented MNA to successfully penetrate into the 10% agarose gel indicates that, for the current design, alternative photomaterials should be used for *es*DLW-based printing. In particular, researchers have reported DLW-compatible fused silica glass-based photo-materials,^[87]^ which are now available commercially and would provide an order of magnitude increase in *E* of the fabricated MNAs. Alternatively, while we designed each microneedle with 10-μm-thick walls and 50 μm ODs, both dimensions could be readily increased to improve the mechanical strength. For excised mouse brains specifically, the ex vivo investigations in the current study revealed effective MNA-mediated penetration, microinjection, and retraction operations without any instances of microneedle-associated mechanical failures (e.g., buckling or fracture). In addition, throughout both in vitro microfluidic cyclic burst-pressure characterizations (with applied pressures in excess of 250 kPa) and ex vivo brain tissue experiments, the MNA-capillary interface exhibited consistent fluidic integrity, without any signs of undesired leakage phenomena or MNA detachment from the capillary.

We envision that future efforts could extend the methodology reported here to achieve novel MNA designs that remediate the deficits of single-needle injection strategies by expanding the delivery range via simultaneous, distributed microinjection. For example, as both in vitro and ex vivo experiments for MNA-mediated microinjections of nanoparticle suspensions revealed homogeneous distributions of implanted particles, such capabilities could offer new means to address the cell crowding challenges of SCT associated with single-needle delivery systems that contribute to low cell viability and, thus, limited therapeutic efficacy.^[88–91]^ Such a pathway to improved SCT could hold distinctive promise for treating a diversity of medical conditions and neurodegenerative diseases, but further studies are needed to explore the potential for MNAs at this scale to enhance therapies that rely on fluidic microinjections—not only for stem cells, but also additional therapeutic payloads (e.g., growth factors and viruses for gene therapy)—into the brain. Nonetheless, given the vast diversity of scientific and clinical applications that are founded on microinjections and/or microneedles, the presented hybrid additive manufacturing strategy offers unique potential as an enabling technology for realizing entirely new classes of MNAs to advance scientific discovery and promote human health and well-being.

## 4. Experimental Section

### Batch Capillary Array Fabrication via DLP 3D Printing:

The computer-aided design (CAD) software, SolidWorks (Dassault Systèmes, France), was used to generate models of batch arrays of capillaries (Figure S1, Supporting Information). Models were exported as STL files and then imported into the CAM (slicer) software for the Miicraft M50 DLP 3D printer (CADworks3D, Canada) to define the print parameter settings (Table S1, Supporting Information). The batch capillary arrays were DLP-printed using Clear Microfluidics Resin V7.0a (CAdworks3D) with the layer height set to 50 μm. Following the DLP printing process, the build plate was removed and the prints were manually detached from the build plate using a razor blade. The prints were developed in methanol for ≈10 s and then methanol was perfused through each capillary to eliminate any residual resin from the interiors. After one additional rinse with methanol, the prints were washed with 90% isopropyl alcohol (IPA). The prints were then dried with pressurized air and postcured under UV light for 20 s (flipping the device after 10 s to cure both sides equally).

### MNA Fabrication Atop the Capillaries via esDLW:

The microneedle arrays—modeled using SolidWorks (Dassault Systèmes)—were designed with identical needles (ID = 30 μm; OD = 50 μm; height = 550 μm) and arrayed with 100 μm needle-to-needle spacing (Figure S3, Supporting Information). MNA models were exported as STL files and then imported into the CAM software, DeScribe (Nanoscribe), to define the print parameter settings (Table S2, Supporting Information), which included a hatching distance of 800 nm and a layer height of 2.5 μm. Initially, IP-Q photoresist (Nanoscribe) was dispensed directly atop the DLP-printed capillaries and the batch was then loaded into the Nanoscribe Photonic Professional GT2 DLW 3D printer (Figure S2, Supporting Information). For *es*DLW printing, the dip-in laser lithography (DiLL) mode was used with a 10× objective lens, a laser power of 27.5 mW, and a laser scanning speed of 120 000 μm s^−1^. The printing process was initiated with 50 μm of overlap with the top capillary surfaces. Following the *es*DLW process, the batch assembly (with MNAs printed atop the capillaries) was removed from the DLW printer for development. The prints were developed using propylene glycol monomethyl ether acetate (PGMEA) for 30 min and IPA for 5 min, and then dried using a gentle stream of N_2_ gas. Individual MNA-capillary assemblies were removed from the batch by manually severing the five connecting structures arrayed radially around each capillary (Movie S3, Supporting Information).

### Finite Element Analysis (FEA):

Numerical simulations of the MNA compression test were performed using the commercially available software, ABAQUS/Standard (Abaqus Inc., Palo Alto, CA). Initially, the complete 3D CAD model of the MNA (i.e., including both the base and needles) was imported into the FEA software, and then the distinct material properties were set. Specifically, the MNA was modeled as a linear elastic homogeneous material (*E* = 250 MPa; *v* = 0.49). The mesh was constructed using four-node, linear, 3D-stress-tetrahedra elements (ABAQUS element type C3D4H), and the accuracy was verified by mesh convergence. During all studies, the circular bottom surface orthogonal to the loading direction was modeled to be perfectly fixed. A static analysis (*STATIC step with NLGEOM = ON in ABAQUS) was conducted to characterize the nonlinear response and loaded the structure by linearly increasing the applied tip force. To characterize the nonlinear response at the interface between the needle tips and the brain substrate, the bottom surface of the cylinder mimicking the brain sample was modeled to be fully clamped while a displacement was applied to the MNA’s cylindrical base. A surface-to-surface contact was defined between the brain substrate and the MNA needle tips. Both tangential and normal contact behaviors were defined. The MNA was modeled as a linear elastic homogeneous material, while the brain substrate was modeled as a hyperelastic Neo-Hookean material. To characterize the nonlinear response at the interface between the needle tips and the brain substrate, a dynamic implicit analysis (*DYNAMIC step with NLGEOM = ON in ABAQUS) was conducted.

### MNA Mechanical Characterization:

Mechanical testing on the MNAs was conducted using a Q800 Dynamic Mechanical Analysis (DMA) system (TA Instruments, New Castle, DE) equipped with a compression clamp. Samples were compressed at a rate of 0.1 N min^−1^ until the failure was confirmed via optical microscopy. Values for *E* and *σγ* of MNAs were calculated from the linear region of the resulting stress-strain curve. To evaluate the puncture ability of the MNAs, hydrogels with different stiffness were prepared by mixing agarose gel powder in 1% PBS (Sigma-Aldrich, Saint Louis, MO) at four different concentration levels: 1.2%, 2.4%, 5%, and 10%. The solutions were heated to a boiling temperature and then cooled down until the hydrogels were set at room temperature. Before each MNA puncture, the top surface of the hydrogel was rinsed with PBS. The MNA was mounted on a stereotaxic manipulator, slowly inserted into the hydrogel samples, and optically monitored for any signs of failure.

### Microfluidic Cyclic Burst-Pressure Experimentation:

Microfluidic testing was performed using a Fluigent Microfluidic Control System and flow-rate platform coupled with OxyGEN software (Fluigent, France). DI water was inputted via fluidic tubing and stainless-steel catheter couplers (20G, Instech, Plymouth Meeting, PA) connected to the opposing end of the capillaries (i.e., without a printed MNA). Three separate sets of cyclic burst-pressure experiments were performed corresponding to applied pressures programmed at 100, 200, or 300 kPa for 2 s, but then set to 0 kPa for 2 s after each pressure increase for all cases. Each set of experiments was performed for 100 cycles while the MNA-capillary interface was optically monitored using an inverted microscope (Motic) connected to a charge-coupled device (CCD) camera (Motic). Both input pressure and flow-rate data were recorded using the Fluigent system. All experiments were conducted under room temperature environment (20–25 °C).

### Ex Vivo Mouse Brain Extraction and Experimentation:

Brain tissues excised from 6-month-old male mice (Wild-type C57BL/6 J, Jackson Laboratory) were used for all ex vivo experiments. Each brain with an intact dura mater was excised within 10 min of euthanasia and stored in cold PBS on ice prior to testing. To maintain tissue integrity, the tissue samples were handled gently before and during the experiment. Each MNA-capillary assembly was interfaced with a custom-built nanoinjector (Narishige) and mounted on a stereotax with a digital display (#68807, RWD, China) to control the displacement and perform microinjections. In separate experiments, blue-dyed water and green fluorescent nanoparticles (505/515, 100 nm diameter, #F8803, Thermofisher) diluted with PBS were injected into the freshly dissected mouse cerebral cortex (or agarose gel) using either an MNA-capillary assembly connected to a micromanipulator (#MO10, Narishige) or a Hamilton syringe with a 33G needle connected to a motorized pump (#78–8130, KD Scientific, Holliston, MA). The injection depth was 500 μm with an extra 200 μm overshoot. The injection duration was ≈2 min for both MNA and Hamilton syringe-mediated injections. After injection with fluorescent nanoparticles, the fresh mouse brains were fixed with 4% paraformaldehyde for 2 d, rinsed, and mounted on glass slides for imaging under a two-photon microscope. These studies were performed in accordance with the National Institutes of Health (NIH) Guide for Care and Use of Laboratory Animals and the University of Maryland, School of Medicine, Animal Care and Use Committee.

### Optical Characterizations:

SEM images were captured using a TM4000 Tabletop SEM (Hitachi, Tokyo, Japan) under low vacuum, which allowed for imaging of uncoated samples. The mechanical tests were recorded using a Monocular Max 300× microscope objective and a 41MP USB C-Mount Industry Microscope Camera Set (Hayear Electronics Co. Ltd., Shenzhen, China). Brightfield microscopy during microfluidic testing was performed using an inverted microscope (Motic AE31, Motic, Canada) connected to a CCD camera (Moticam Pro 285B, Motic). For ex vivo microinjection experiments, the injection process was recorded using the Monocular microscope while the fluorescent images of the top view of the gel injection site were captured using a DMi8 automated fluorescence microscope (Leica Microsystems, Wetzlar, Germany). The 3D stack images of the injection sites were acquired using the Modular In Vivo Multiphoton Microscopy System designed by Janelia Research Campus, Howard Hughes Medical Institute. A 900 nm laser (≈5 mW) was used for excitation of the green fluorescent nanoparticles. The 3D stacks from the top of the brain to the bottom of the needle track were acquired at a step size of 2 μm under a water-immersion 25× objective (numerical aperture of 1.05, Olympus). Fluorescence emission was collected by two GaAsP photomultiplier tubes after being split by a dichroic mirror (560 nm, T560pxrxt, Chroma) with an emission filter green (510/84 nm, 84–097, Edmund) fluorescence. A similar acquisition setting was used for imaging the needle tracks in hydrogels injected with the fluorescent nanoparticles. Fluorescence images were processed and visualized with ImageJ (NIH, Bethesda, MD). BigDataViewer was used to adjust the tilting angle of the 3D stack for optimized visualization. For comparisons of needle-to-needle injection sites within the MNAs as well as injection distributions between the MNA and Hamilton injections, ImageJ was used to quantify the fluorescence intensities.

### Statistical Analysis:

Statistical significance was quantified via unpaired Student’s *t*-tests, with two-tailed *p* values greater than 0.05 considered statistically indistinguishable. A minimum of three samples were used to quantify any means reported, with data presented in the text as mean ± standard deviation (*S.D.*).

## Supplementary Material

Supp. Material

Movie 1

Movie 2

Movie 3

Movie 4

Movie 5

Movie 6

Movie 7

Movie 8

Movie 9

Movie 10

## Data Availability

The data that support the findings of this study are available from the corresponding author upon reasonable request.

## References

[1] Yang B, Kong J, Fang X, Nat. Commun 2022, 13, 3999. 35810160 10.1038/s41467-022-31740-3 PMC9271037

[2] Yang Y, Xu L, Jiang D, Chen BZ, Luo R, Liu Z, Qu X, Wang C, Shan Y, Cui Y, Zheng H, Wang Z, Wang ZL, Guo XD, Li Z, Adv. Funct. Mater 2021, 31, 2104092.

[3] Yang J, Yang J, Gong X, Zheng Y, Yi S, Cheng Y, Li Y, Liu B, Xie X, Yi C, Jiang L, Adv. Healthcare Mater 2022, 11, 2102547. 10.1002/adhm.202102547 35034429

[4] Park W, Nguyen VP, Jeon Y, Kim B, Li Y, Yi J, Kim H, Leem JW, Kim YL, Kim DR, Paulus YM, Lee CH, Sci. Adv 2022, 8, eabn1772. 35353558 10.1126/sciadv.abn1772 PMC8967230

[5] Kusama S, Sato K, Matsui Y, Kimura N, Abe H, Yoshida S, Nishizawa M, Nat. Commun 2021, 12, 658. 33510169 10.1038/s41467-021-20948-4 PMC7843990

[6] Lin L, Wang Y, Cai M, Jiang X, Hu Y, Dong Z, Yin D, Liu Y, Yang S, Liu Z, Zhuang J, Xu Y, Guo CF, Chang L, Adv. Funct. Mater 2022, 32, 2109187.

[7] Yadav V, Sharma PK, Murty US, Mohan NH, Thomas R, Dwivedy SK, Banerjee S, Int. J. Pharm 2021, 605, 120815. 34153441 10.1016/j.ijpharm.2021.120815

[8] Makvandi P, Maleki A, Shabani M, Hutton ARJ, Kirkby M, Jamaledin R, Fang T, He J, Lee J, Mazzolai B, Donnelly RF, Tay FR, Chen G, Mattoli V, Matter 2022, 5, 390.

[9] Ali M, Namjoshi S, Benson HAE, Mohammed Y, Kumeria T, J. Controlled Release 2022, 347, 561. 10.1016/j.jconrel.2022.04.043 35525331

[10] Vora LK, Moffatt K, Tekko IA, Paredes AJ, Volpe-Zanutto F, Mishra D, Peng K, Raj Singh Thakur R, Donnelly RF, Eur. J. Pharm. Biopharm 2021, 159, 44. 33359666 10.1016/j.ejpb.2020.12.006

[11] Li WX, Zhang XP, Chen BZ, Fei WM, Cui Y, Zhang CY, Guo XD, Drug Delivery Transl. Res 2022, 12, 2275. 10.1007/s13346-021-01113-2 35112330

[12] Mcmillan CLD, Choo JJY, Idris A, Supramaniam A, Modhiran N, Amarilla AA, Isaacs A, Cheung STM, Liang B, Bielefeldt-Ohmann H, Azuar A, Acharya D, Kelly G, Fernando GJP, Landsberg MJ, Khromykh AA, Watterson D, Young PR, Mcmillan NAJ, Muller DA, Sci. Adv 2021, 7, eabj8065. 34714668 10.1126/sciadv.abj8065 PMC8555896

[13] Zhang X, Fu X, Chen G, Wang Y, Zhao Y, Adv. Sci 2021, 8, 2101210. 10.1002/advs.202101210 PMC8425882 34218532

[14] Abe H, Sato K, Kimura N, Kusama S, Inoue D, Yamasaki K, Nishizawa M, Adv. Nanobiomed. Res 2022, 2, 2100066.

[15] Li Y, Zhang H, Yang R, Laffitte Y, Schmill U, Hu W, Kaddoura M, Blondeel EJM, Cui B, Microsyst. Nanoeng 2019, 5, 41. 31636931 10.1038/s41378-019-0077-y PMC6799813

[16] Cárcamo-Martínez Á, Mallon B, Domínguez-Robles J, Vora LK, Anjani QK, Donnelly RF, Int. J. Pharm 2021, 599, 120455. 33676993 10.1016/j.ijpharm.2021.120455

[17] Li R, Liu X, Yuan X, Wu S, Li L, Jiang X, Li B, Jiang X, Gou M, Int. J. Bioprint 2022, 8, 553. 35669318 10.18063/ijb.v8i2.553 PMC9159536

[18] Chen Z, Ren L, Li J, Yao L, Chen Y, Liu B, Jiang L, Acta Biomater. 2018, 65, 283. 29107057 10.1016/j.actbio.2017.10.030

[19] Dervisevic M, Alba M, Yan L, Senel M, Gengenbach TR, Prieto-Simon B, Voelcker NH, Adv. Funct. Mater 2022, 32, 2009850.

[20] Roh H, Yoon YJ, Park JS, Kang D-H, Kwak SM, Lee BC, Im M, Nano-Micro Lett. 2021, 14, 24. 10.1007/s40820-021-00778-1 PMC8656445 34888758

[21] Chen G, Zhang Y, Li C, Huang D, Wang Q, Wang Q, Adv. Healthcare Mater 2018, 7, 1800497. 10.1002/adhm.201800497 30019509

[22] Lee AS, Inayathullah M, Lijkwan MA, Zhao X, Sun W, Park S, Hong WX, Parekh MB, Malkovskiy AV, Lau E, Qin X, Pothineni VR, Sanchez-Freire V, Zhang WY, Kooreman NG, Ebert AD, Chan CKF, Nguyen PK, Rajadas J, Wu JC, Nat. Biomed. Eng 2018, 2, 104. 29721363 10.1038/s41551-018-0191-4 PMC5927627

[23] Clark AY, Martin KE, García JR, Johnson CT, Theriault HS, Han WM, Zhou DW, Botchwey EA, García AJ, Nat. Commun 2020, 11, 114. 31913286 10.1038/s41467-019-14000-9 PMC6949269

[24] Espuny-Camacho I, Michelsen KA, Linaro D, Bilheu A, Acosta-Verdugo S, Herpoel A, Giugliano M, Gaillard A, Vanderhaeghen P, Cell Rep. 2018, 23, 2732. 29847802 10.1016/j.celrep.2018.04.094 PMC5990494

[25] Palma-Tortosa S, Tornero D, Grønning Hansen M, Monni E, Hajy M, Kartsivadze S, Aktay S, Tsupykov O, Parmar M, Deisseroth K, Skibo G, Lindvall O, Kokaia Z, Proc. Natl. Acad. Sci. U. S. A 2020, 117, 9094. 32253308 10.1073/pnas.2000690117 PMC7183146

[26] Liang Y, Walczak P, Bulte JWM, Biomaterials 2013, 34, 5521. 23623429 10.1016/j.biomaterials.2013.03.095 PMC3653424

[27] Othman FA, Tan SC, Brain Sci. 2020, 10, 893. 33238363 10.3390/brainsci10110893 PMC7700351

[28] Liu H, Reiter S, Zhou X, Chen H, Ou Y, Lenahan C, He Y, Front. Cell. Neurosci 2021, 15, 637210. 33732111 10.3389/fncel.2021.637210 PMC7959708

[29] Jolien De M, Jolien De P, Said H-I, Int. J. Crit. Care Emerg. Med 2018, 4, 058.

[30] Vandamme T, Pharm J. Bioallied Sci. 2014, 6, 2. 10.4103/0975-7406.124301 PMC3895289 24459397

[31] Lerch JP, Carroll JB, Dorr A, Spring S, Evans AC, Hayden MR, Sled JG, Henkelman RM, NeuroImage 2008, 41, 243. 18387826 10.1016/j.neuroimage.2008.02.019

[32] Cui H, Yao D, Hensleigh R, Lu H, Calderon A, Xu Z, Davaria S, Wang Z, Mercier P, Tarazaga P, Zheng XR, Science 2022, 376, 1287. 35709267 10.1126/science.abn0090

[33] Taylor JM, Luan H, Lewis JA, Rogers JA, Nuzzo RG, Braun PV, Adv. Mater 2022, 34, 2108391. 10.1002/adma.202108391 35233865

[34] Hubbard JD, Acevedo R, Edwards KM, Alsharhan AT, Wen Z, Landry J, Wang K, Schaffer S, Sochol RD, Sci. Adv 2021, 7, eabe5257. 34261646 10.1126/sciadv.abe5257 PMC8279518

[35] Derakhshandeh H, Aghabaglou F, Mccarthy A, Mostafavi A, Wiseman C, Bonick Z, Ghanavati I, Harris S, Kreikemeier-Bower C, Moosavi Basri SM, Rosenbohm J, Yang R, Mostafalu P, Orgill D, Tamayol A, Adv. Funct. Mater 2020, 30, 1905544. 34354556 10.1002/adfm.201905544 PMC8336080

[36] Samandari M, Aghabaglou F, Nuutila K, Derakhshandeh H, Zhang Y, Endo Y, Harris S, Barnum L, Kreikemeier-Bower C, Arab-Tehrany E, Peppas NA, Sinha I, Tamayol A, Adv. Healthcare Mater 2021, 10, 2001800. 10.1002/adhm.202001800 33586339

[37] Yin M, Xiao L, Liu Q, Kwon S-Y, Zhang Y, Sharma PR, Jin L, Li X, Xu B, Adv. Healthcare Mater 2019, 8, 1901170. 10.1002/adhm.201901170 PMC6918473 31664794

[38] Hwang HH, Zhu W, Victorine G, Lawrence N, Chen S, Small Methods 2018, 2, 1700277. 30090851 10.1002/smtd.201700277 PMC6078427

[39] Wu M, Zhang Y, Huang H, Li J, Liu H, Guo Z, Xue L, Liu S, Lei Y, Mater. Sci. Eng., C 2020, 117, 111299. 10.1016/j.msec.2020.111299 32919660

[40] Economidou SN, Uddin MJ, Marques MJ, Douroumis D, Sow WT, Li H, Reid A, Windmill JFC, Podoleanu A, Addit. Manuf 2021, 38, 101815.

[41] Chen Z, Wu H, Zhao S, Chen X, Wei T, Peng H, Chen Z, Mol. Pharmaceutics 2022, 19, 3314. 10.1021/acs.molpharmaceut.2c00466 35947780

[42] Xenikakis I, Tsongas K, Tzimtzimis EK, Zacharis CK, Theodoroula N, Kalogianni EP, Demiri E, Vizirianakis IS, Tzetzis D, Fatouros DG, Int. J. Pharm 2021, 597, 120303. 33540009 10.1016/j.ijpharm.2021.120303

[43] Alsharhan AT, Acevedo R, Warren R, Sochol RD, Lab Chip 2019, 19, 2799. 31334525 10.1039/c9lc00542k

[44] Han JY, Warshawsky S, Devoe DL, Sci. Rep 2021, 11, 10980. 34040116 10.1038/s41598-021-90571-2 PMC8155204

[45] Restaino M, Eckman N, Alsharhan AT, Lamont AC, Anderson J, Weinstein D, Hall A, Sochol RD, Adv. Mater. Technol 2021, 6, 2100222.

[46] Acevedo R, Restaino MA, Yu D, Hoag SW, Flank S, Sochol RD,J. Microelectromech. Syst 2020, 29, 924.

[47] Ziesmer J, Tajpara P, Hempel N-J, Ehrström M, Melican K, Eidsmo L, Sotiriou GA, Adv. Mater. Technol 2021, 6, 2001307. 34307835 10.1002/admt.202001307 PMC8281827

[48] Balmert SC, Carey CD, Falo GD, Sethi SK, Erdos G, Korkmaz E, Falo LD, J. Controlled Release 2020, 317, 336. 10.1016/j.jconrel.2019.11.023 PMC8237702 31756393

[49] Cárcamo-MartínezÁ, MallonB, Domínguez-RoblesJ, CordeiroAS, CelentanoM, LarrañetaE, BellSEJ, DonnellyRF,J. Mater. Chem. B 2020, 8, 5425.3249047310.1039/d0tb00962h

[50] Ebrahiminejad V, Faraji Rad Z, Prewett PD, Davies GJ, Beilstein J Nanotechnol. 2022, 13, 629. 10.3762/bjnano.13.55 PMC9273988 35874440

[51] Tekko IA, Vora LK, Volpe-Zanutto F, Moffatt K, Jarrahian C, Mccarthy HO, Donnelly RF, Adv. Funct. Mater 2022, 32, 2106999.

[52] Battisti M, Vecchione R, Casale C, Pennacchio FA, Lettera V, Jamaledin R, Profeta M, Di Natale C, Imparato G, Urciuolo F, Netti PA, Front. Bioeng. Biotechnol 2019, 7, 296. 31781550 10.3389/fbioe.2019.00296 PMC6856554

[53] Faraji Rad Z, Prewett PD, Davies GJ, Addit. Manuf 2022, 56, 102953.

[54] Faraji Rad Z, Prewett PD, Davies GJ, Manuf. Lett 2021, 30, 39.

[55] Faraji Rad Z, Nordon RE, Anthony CJ, Bilston L, Prewett PD, Arns J-Y, Arns CH, Zhang L, Davies GJ, Microsyst. Nanoeng 2017, 3, 17034. 31057872 10.1038/micronano.2017.34 PMC6445010

[56] Alsharhan AT, Young OM, Xu X, Stair AJ, Sochol RD, J. Micromech. Microeng 2021, 31, 044001.

[57] Lamont AC, Alsharhan AT, Sochol RD, Sci. Rep 2019, 9, 394. 30674934 10.1038/s41598-018-36727-z PMC6344532

[58] Moussi K, Bukhamsin A, Hidalgo T, Kosel J, Adv. Eng. Mater 2020, 22, 1901358.

[59] Szeto B, Aksit A, Valentini C, Yu M, Werth EG, Goeta S, Tang C, Brown LM, Olson ES, Kysar JW, Lalwani AK, Hear. Res 2021, 400, 108141. 33307286 10.1016/j.heares.2020.108141 PMC8656365

[60] Moussi K, Haneef AA, Alsiary RA, Diallo EM, Boone MA, Abu-Araki H, Al-Radi OO, Kosel J, Adv. Mater. Technol 2021, 6, 2100037.

[61] Trautmann A, Roth G-L, Nujiqi B, Walther T, Hellmann R, Microsyst. Nanoeng 2019, 5, 6. 31057933 10.1038/s41378-019-0046-5 PMC6387975

[62] Lim SH, Kathuria H, Amir MHB, Zhang X, Duong HTT, Ho PC-L, Kang L, J. Controlled Release 2021, 329, 907. 10.1016/j.jconrel.2020.10.021 33068646

[63] Barbot A, Power M, Seichepine F, Yang G-Z, Sci. Adv 2020, 6, eaba5660. 32518828 10.1126/sciadv.aba5660 PMC7253165

[64] Barbot A, Wales D, Yeatman E, Yang G-Z, Adv. Sci 2021, 8, 2004643. 10.1002/advs.202004643 PMC8132067 34026456

[65] Acevedo R, Wen Z, Rosenthal IB, Freeman EZ, Restaino M, Gonzalez N, Sochol RD, in 2021 IEEE 34th Int. Conf. Micro Electro Mechan. Syst. (MEMS), 2021, pp. 10–13. 10.1109/MEMS51782.2021.9375347.

[66] Makvandi P, Kirkby M, Hutton ARJ, Shabani M, Yiu CKY, Baghbantaraghdari Z, Jamaledin R, Carlotti M, Mazzolai B, Mattoli V, Donnelly RF, Nano-Micro Lett. 2021, 13, 93. 10.1007/s40820-021-00611-9 PMC8006208 34138349

[67] Abalymov A, Parakhonskiy B, Skirtach A, Polymers 2020, 12, 620. 32182751 10.3390/polym12030620 PMC7182904

[68] Saveleva MS, Eftekhari K, Abalymov A, Douglas TEL, Volodkin D, Parakhonskiy BV, Skirtach AG, Front. Chem 2019, 7, 179. 31019908 10.3389/fchem.2019.00179 PMC6459030

[69] Kim HN, Kang D-H, Kim MS, Jiao A, Kim D-H, Suh K-Y, Ann. Biomed. Eng 2012, 40, 1339. 22258887 10.1007/s10439-012-0510-y PMC5439960

[70] Nemir S, West JL, Ann. Biomed. Eng 2010, 38, 2. 19816774 10.1007/s10439-009-9811-1

[71] Gissibl T, Thiele S, Herkommer A, Giessen H, Nat. Photonics 2016, 10, 554. 10.1038/ncomms11763 PMC4931017 27339700

[72] Dietrich P-I, Blaicher M, Reuter I, Billah M, Hoose T, Hofmann A, Caer C, Dangel R, Offrein B, Troppenz U, Moehrle M, Freude W, Koos C, Nat. Photonics 2018, 12, 241.

[73] Power M, Thompson AJ, Anastasova S, Yang G-Z, Small 2018, 14, 1703964. 10.1002/smll.201703964 29479810

[74] Wahlberg B, Ghuman H, Liu JR, Modo M, Sci. Rep 2018, 8, 9194. 29907825 10.1038/s41598-018-27568-x PMC6004017

[75] Ates HC, Nguyen PQ, Gonzalez-Macia L, Morales-Narváez E, Güder F, Collins JJ, Dincer C, Nat. Rev. Mater 2022, 7, 887. 35910814 10.1038/s41578-022-00460-x PMC9306444

[76] Lin S, Cheng X, Zhu J, Wang B, Jelinek D, Zhao Y, Wu T-Y, Horrillo A, Tan J, Yeung J, Yan W, Forman S, Coller HA, Milla C, Emaminejad S, Sci. Adv 2022, 8, eabq4539. 36149955 10.1126/sciadv.abq4539 PMC9506728

[77] Tehrani F, Teymourian H, Wuerstle B, Kavner J, Patel R, Furmidge A, Aghavali R, Hosseini-Toudeshki H, Brown C, Zhang F, Mahato K, Li Z, Barfidokht A, Yin L, Warren P, Huang N, Patel Z, Mercier PP, Wang J, Nat. Biomed. Eng 2022, 6, 1214. 35534575 10.1038/s41551-022-00887-1

[78] Lee Y, Kang T, Cho HR, Lee GJ, Park OK, Kim S, Lee B, Kim HM, Cha GD, Shin Y, Lee W, Kim M, Kim H, Song YM, Choi SH, Hyeon T, Kim D-H, Adv. Mater 2021, 33, 2100425. 10.1002/adma.202100425 33955598

[79] Economidou SN, Douroumis D, Adv. Drug Delivery Rev 2021, 173, 60. 10.1016/j.addr.2021.03.007 33775705

[80] Chen W, Wainer J, Ryoo SW, Qi X, Chang R, Li J, Lee SH, Min S, Wentworth A, Collins JE, Tamang S, Ishida K, Hayward A, Langer R, Traverso G, Sci. Adv 2022, 8, eabk1792. 34985942 10.1126/sciadv.abk1792 PMC8730401

[81] Gernhardt M, Truong VX, Barner-Kowollik C, Adv. Mater 2022, 34, 2203474. 10.1002/adma.202203474 35918791

[82] Accardo A, Blatché M-C, Courson R, Loubinoux I, Vieu C, Malaquin L, Mater. Today 2018, 21, 315. 10.1002/smll.201700621 28558136

[83] Tumbleston JR, Shirvanyants D, Ermoshkin N, Janusziewicz R, Johnson AR, Kelly D, Chen K, Pinschmidt R, Rolland JP, Ermoshkin A, Samulski ET, Desimone JM, Science 2015, 347, 1349. 25780246 10.1126/science.aaa2397

[84] Kelly BE, Bhattacharya I, Heidari H, Shusteff M, Spadaccini CM, Taylor HK, Science 2019, 363, 1075. 30705152 10.1126/science.aau7114

[85] Loterie D, Delrot P, Moser C, Nat. Commun 2020, 11, 852. 32051409 10.1038/s41467-020-14630-4 PMC7015946

[86] Regehly M, Garmshausen Y, Reuter M, König NF, Israel E, Kelly DP, Chou C-Y, Koch K, Asfari B, Hecht S, Nature 2020, 588, 620. 33361791 10.1038/s41586-020-3029-7

[87] Kotz F, Quick AS, Risch P, Martin T, Hoose T, Thiel M, Helmer D, Rapp BE, Adv. Mater 2021, 33, 2006341. 10.1002/adma.202006341 PMC11469267 33448090

[88] Lee S, Choi E, Cha M-J, Hwang K-C, Oxid. Med. Cell. Longevity 2015, 2015, 632902. 10.1155/2015/632902 PMC4333334 25722795

[89] Barker RA, Dunnett SB, Faissner A, Fawcett JW, Exp. Neurol 1996, 141, 79. 8797670 10.1006/exnr.1996.0141

[90] Kallur T, Darsalia V, Lindvall O, Kokaia Z, J. Neurosci. Res 2006, 84, 1630. 17044030 10.1002/jnr.21066

[91] Danielyan L, Schwab M, Siegel G, Brawek B, Garaschuk O, Asavapanumas N, Buadze M, Lourhmati A, Wendel H-P, Avci-Adali M, Krueger MA, Calaminus C, Naumann U, Winter S, Schaeffeler E, Spogis A, Beer-Hammer S, Neher JJ, Spohn G, Kretschmer A, Krämer-Albers E-M, Barth K, Lee HJ, Kim SU, Frey WH, Claussen CD, Hermann DM, Doeppner TR, Seifried E, Gleiter CH, , EBioMedicine 2020, 60, 102989. 32920368 10.1016/j.ebiom.2020.102989 PMC7494685

[92] Alsharhan AT, Acevedo R, Warren R, Sochol RD, (2019), 3D microfluidics via cyclic olefin polymer-based in situ direct laser writing. Lab on a Chip, 19(17), 2799–2810. 10.1039/c9lc00542k. 31334525

