# Supplementary material for: 3D-Printed Microinjection Needle Arrays via a Hybrid DLP-Direct Laser Writing Strategy
Source: Adv Mater Technol. Author manuscript; Available in PMC 2023 Apr 14. (PMC10104452; doi:10.1002/admt.202201641)
Supplement: Supp. Material [file NIHMS1872788-supplement-Supp__Material.pdf]

ADVANCED MATERIALS TECHNOLOGIES

Supporting Information

for *Adv. Mater. Technol.,* DOI: 10.1002/admt.202201641

3D-Printed Microinjection Needle Arrays via a Hybrid DLP-Direct Laser Writing Strategy

*Sunandita Sarker, Adira Colton, Ziteng Wen, Xin Xu, Metecan Erdi, Anthony Jones, Peter Kofinas, Eleonora Tubaldi, Piotr Walczak, Miroslaw Janowski, Yajie Liang, and Ryan D. Sochol**

SUPPORTING INFORMATION

3D-Printed Microinjection Needle Arrays *via* a Hybrid DLP-Direct Laser Writing Strategy

Sunandita Sarker[a,b,c], Adira Colton[a,b,c], Ziteng Wen[a], Xin Xu[a], Metecan Erdi[d], Anthony Jones[a,b,c], Peter Kofinas[d], Eleonora Tubaldi[a,b,c], Piotr Walczak[d], Miroslaw Janowski[e], Yajie Liang[e] and Ryan D. Sochol*[abcfg]

a. Department of Mechanical Engineering, University of Maryland, College Park, MD

b. Maryland Robotics Center, University of Maryland, College Park, MD

c. Institute for Systems Research, University of Maryland, College Park, MD

d. Department of Chemical and Biomolecular Engineering, University of Maryland, College Park, MD

e. Program in Image Guided Neurointerventions, Department of Diagnostic Radiology and Nuclear Medicine, University of Maryland School of Medicine, Baltimore, MD

f. Fischell Department of Bioengineering, University of Maryland, College Park, MD

g. Robert E. Fischell Institute for Biomedical Devices, University of Maryland, College Park, MD

* Ryan D. Sochol, 2147 Glenn L. Martin Hall, University of Maryland, College Park, MD 20742, USA; rsochol@umd.edu

Keywords: Additive Manufacturing, 3D Printing, Direct Laser Writing, Digital Light Processing, Microneedles

Supporting Materials:

Figures S1–S6

Tables S1–S2

Movies S1–S10

Supplementary Files

SUPPORTING FIGURES

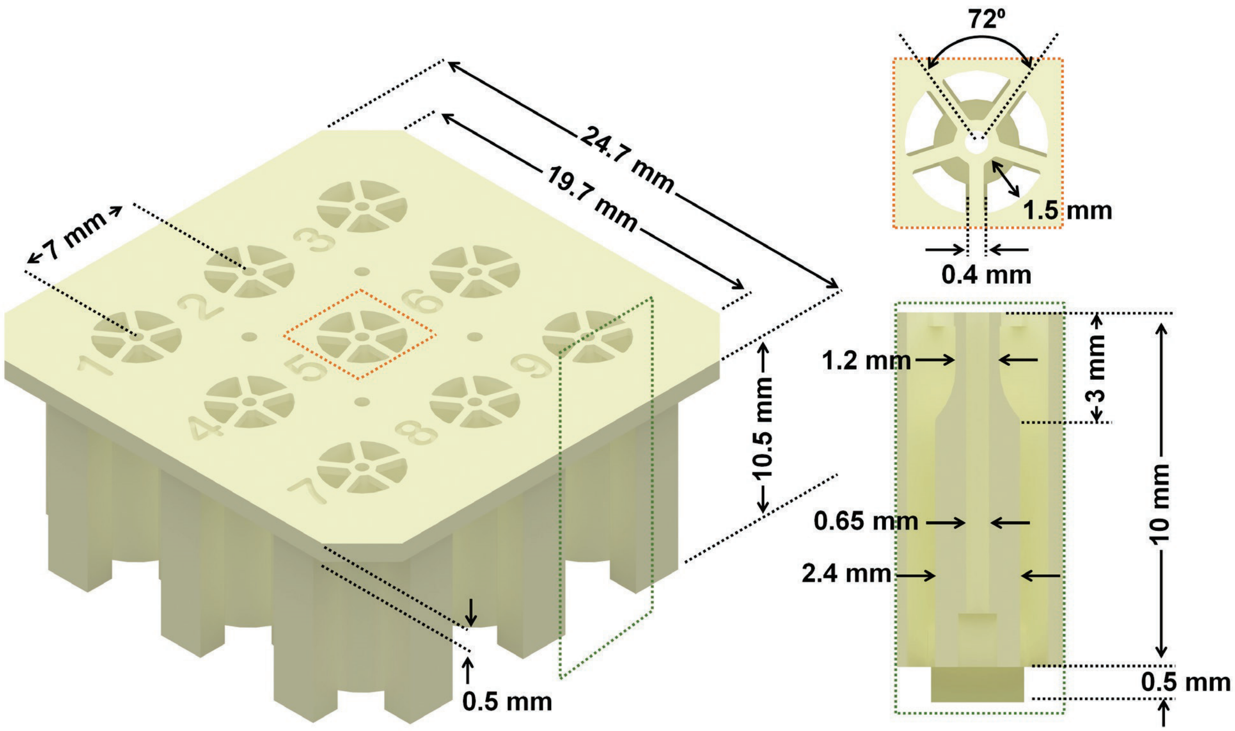

Figure S1 | Design schematic of the batch array of capillaries for Digital Light Processing (DLP)based 3D printing.

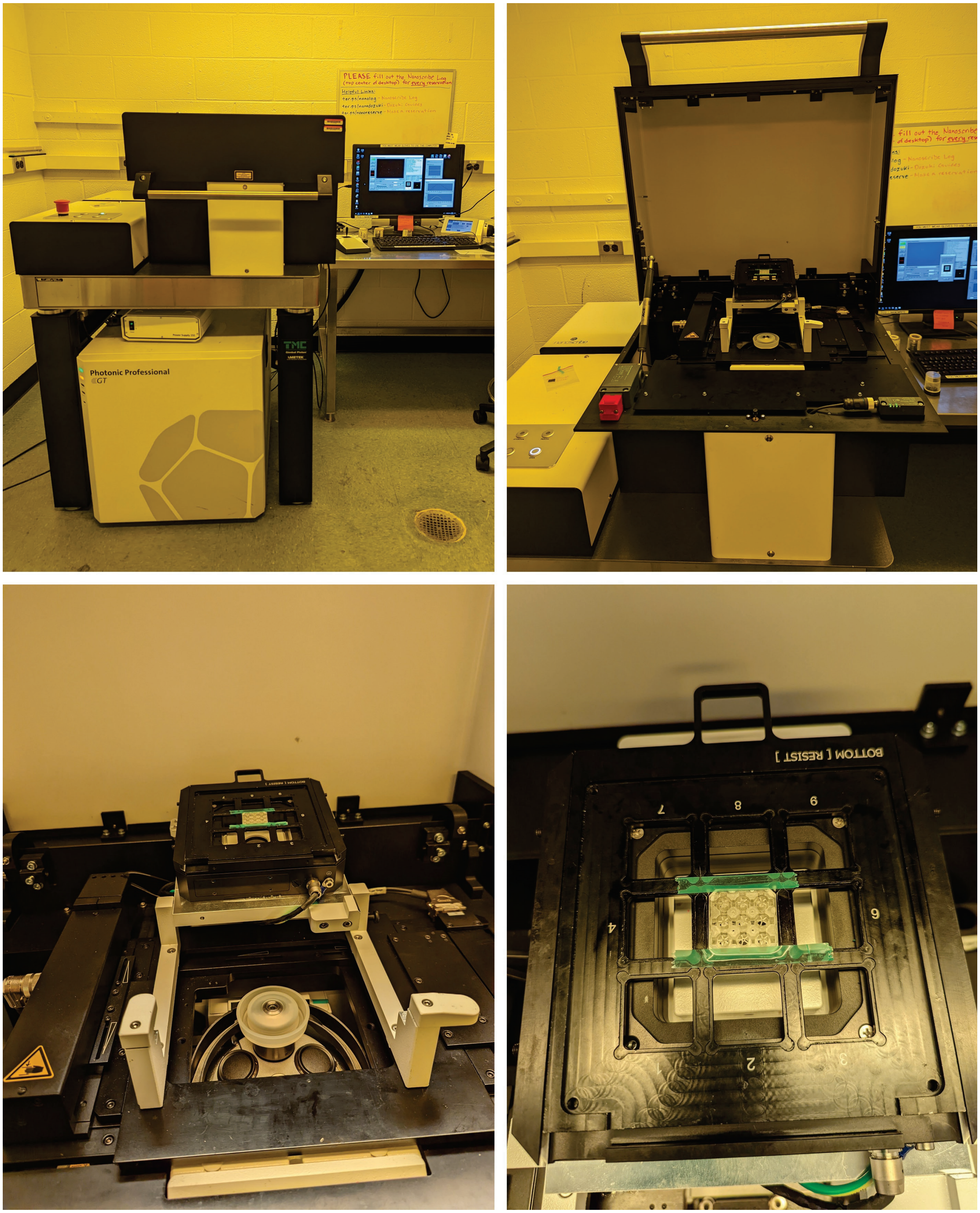

Figure S2 | Images of the Nanoscribe Photonic Professional GT2 Direct Laser Writing (DLW) 3D printer with the DLP-printed batch capillary arrays loaded into the multi-DiLL holder.

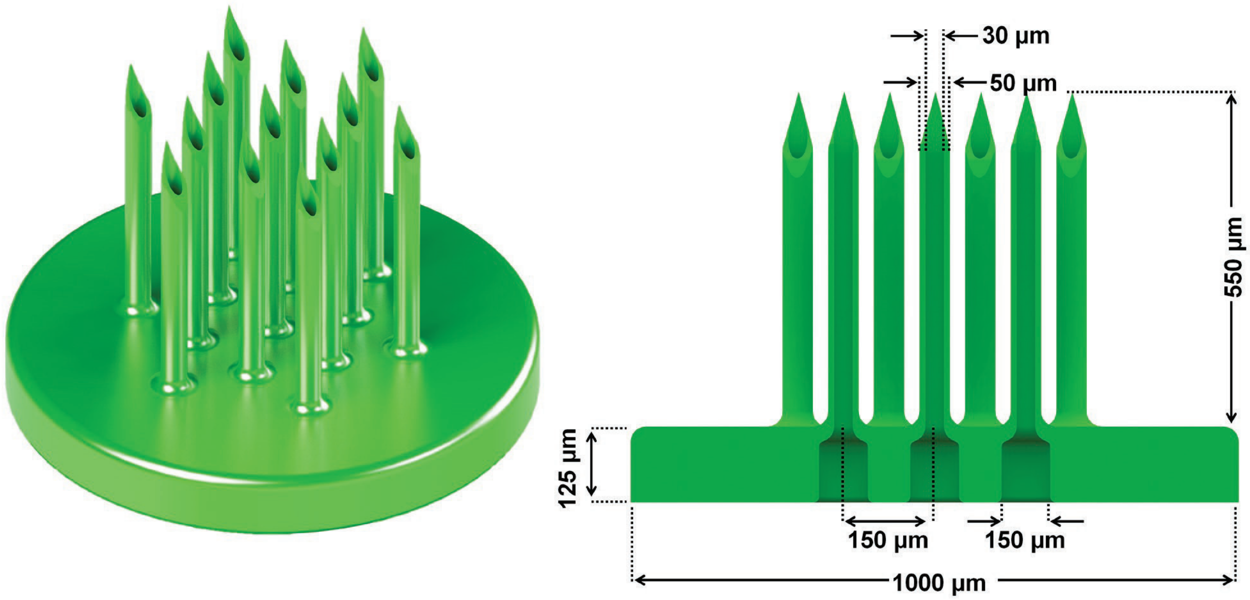

Figure S3 | Design schematic of the microneedle array (MNA) for "*ex situ* DLW (*es*DLW)".

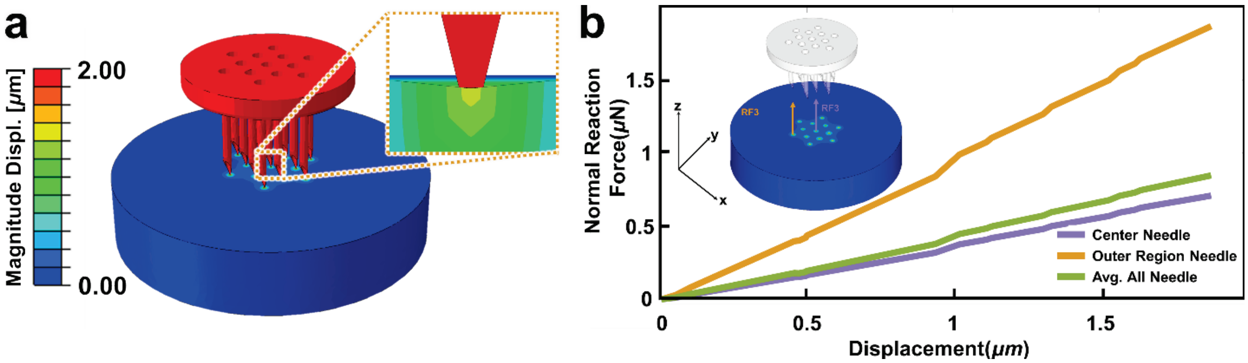

Figure S4 | Examples of Abaqus finite element analysis (FEA) simulations of microneedle-brain tissue micromechanical interactions. **(a)** Deformation of brain tissue substrate at the interface. **(b)** Normal reaction force on arrayed needles.

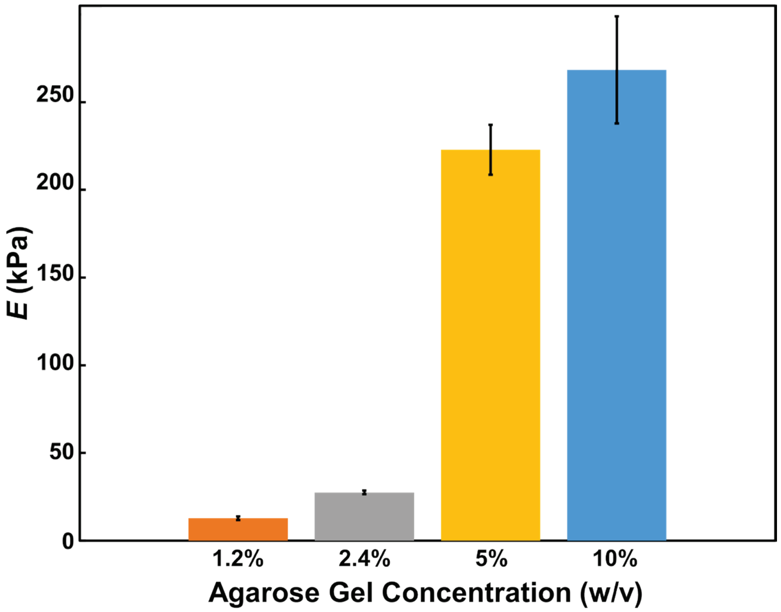

Figure S5 | Experimentally measured *E* for hydrogels of increasing agarose concentration. Error bars denote *S.D.*

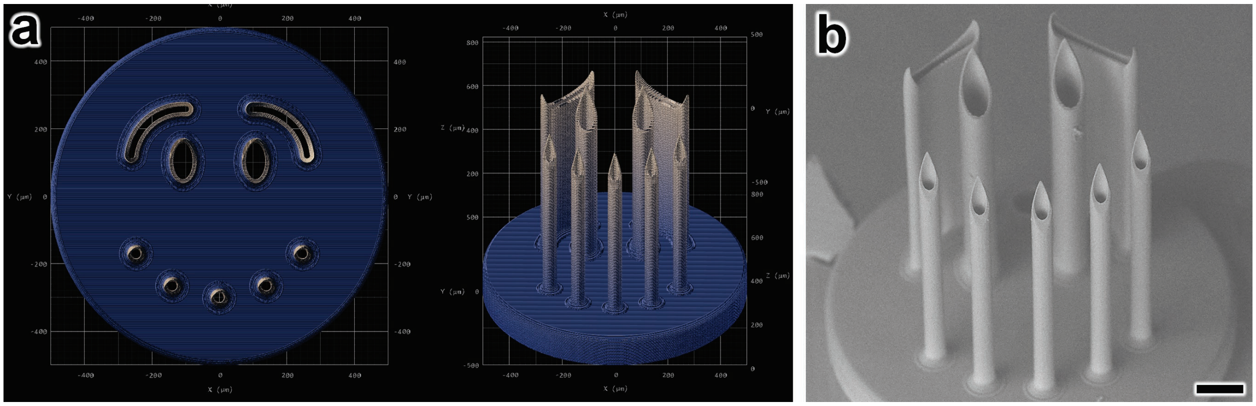

Figure S6 | Demonstrative example of geometric versatility in microneedle-specific design (*e.g.*, size and shape) and array placement. **(a)** CAM simulations of target microneedle array design. **(b)** SEM micrograph of fabrication results. Scale bar = 100 μm.

Table S1. Parameter Settings for Miicraft M50 DLP 3D printer Slicing Tool to 3D print Batch Capillaries

**Parameter**	**Value**
Layer Thickness	50 μm
Layer Exposure time	1.3 s
Base Layer Count	1
Base Layer Exposure Time	25 s
Buffer Layer Count	2

Table S2. Parameter Settings for Describe software to 3D print Microneedle Arrays

**Parameter**	**Value**
Slicing Distance	2.5 μm
Slicing Simplification Tolerance	50 nm
Hatching Distance	800 nm
Hatching Angle	0º
Hatching Angle Offset	90º
Contour Count	5
Contour Distance	0.2 μm
Power Scaling	1.0
Contour Laser Power	90%
Contour Scan Speed	100,000 μm/s
Solid Laser Power	95%
Solid Scan Speed	135,000 μm/s

Supporting Movie Captions

Movie S1 | Two batch arrays of capillaries directly after completion of the 45-min DLP 3D printing process. Video speed = 4×.

Movie S2 | The *es*DLW approach for printing MNAs directly onto DLP-printed capillaries in a single print run. (***Left***) Computer-aided manufacturing (CAM) simulations, and (***Right***) corresponding micrographs of the *es*DLW printing process.

Movie S3 | Protocol for retrieving target MNA-capillary assemblies from the batch by manually severing the five connecting structures.

Movie S4 | Axial compression tests with an *es*DLW-printed MNA.

Movie S5 | MNA puncture and penetration experimental results for hydrogels with agarose concentrations of: (**a**) 1.2%, (**b**) 2.4%, (**c**) 5% and (**d**) 10%.

Movie S6 | Preliminary loading of blue-dyed DI water through the MNA-capillary assembly.

Movie S7 | Video of MNA penetration into and retraction from an excised mouse brain *ex vivo*.

Movie S8 | Video of: (*i*) MNA penetration into an excised mouse brain, (*ii*) MNA-mediated microinjection of blue-dyed DI water into the brain tissue, and then (*iii*) retraction of the MNA from the brain. All operations were performed *ex vivo*.

Movie S9 | Video of nanoparticle microinjection and retraction *in vitro* using 0.6% agarose gel.

Movie S10 | Video of MNA penetration, microinjection, and retraction process for injecting a suspension of fluorescent nanoparticles into brain tissue *ex vivo*.
